# There is no quick, easy, or cheap way to recruit older African Americansto aging and brain health research: *Ten evidence‐based strategies for investing in community engagement*

**DOI:** 10.1002/alz.091673

**Published:** 2025-01-09

**Authors:** Mark A. Gluck, Bernadette A. Fausto, Delores Thornton‐Hammonds, Glenda Wright, Glenn B. Wilson

**Affiliations:** ^1^ Rutgers University–Newark, Newark, NJ USA; ^2^ Pilgrim Baptist Church, Newark, NJ USA

## Abstract

**Background:**

With African Americans having two to three times the prevalence of Alzheimer’s disease (AD) as compared to white Americans, a critical challenge for reducing health disparities is overcoming long‐standing barriers to recruiting African Americans into aging and brain health research. Here, we describe strategies that we have found successful for recruiting over 500 older African Americans from Greater Newark, New Jersey, into brain health and AD research studies.

**Method:**

(1). Build trust through long‐term investment in community health.

(2). Hire a large and varied engagement team with deep connections to community.

(3). Formalize community relationships through a *Community Stakeholders Board*.

(4). Offer frequent event programming and print materials to promote brain health.

(5). Be a resource: Respond to other community needs, even if unrelated to brain health.

(6). Empower, facilitate and recognize community members who embody brain healthy lifestyles.

(7). Recruit older men with programming that addresses men’s interests and affiliations.

(8). Support longitudinal retention through a *Very Important Participants* (VIP) program.

(9). Recognize current research participants as ambassadors for future recruitment.

(10). Communicate scientific results to participants and community members.

**Result:**

These strategies require a sustained and substantial long‐term investment of time, effort, and money, towards building trust and partnerships with local community organizations and community members, with a primary emphasis on creating value for the community and local community‐based and faith‐based organizations, and a secondary consequence of encouraging research participation.

**Conclusion:**

These strategies are readily implementable by other universities doing brain health and Alzheimer’s disease research in underrepresented communities, although they require considerable staffing and financial resources.

